# Raltegravir Cerebrospinal Fluid Concentrations in HIV-1 Infection

**DOI:** 10.1371/journal.pone.0006877

**Published:** 2009-09-01

**Authors:** Aylin Yilmaz, Magnus Gisslén, Serena Spudich, Evelyn Lee, Anura Jayewardene, Francesca Aweeka, Richard W. Price

**Affiliations:** 1 Department of Infectious Diseases, the Sahlgrenska Academy, University of Gothenburg, Gothenburg, Sweden; 2 Department of Neurology, University of California San Francisco, San Francisco, California, United States of America; 3 Department of Pharmacology, University of California San Francisco, San Francisco, California, United States of America; Instituto de Pesquisa Clinica Evandro Chagas, FIOCRUZ, Brazil

## Abstract

**Introduction:**

Raltegravir is an HIV-1 integrase inhibitor currently used in treatment-experienced HIV-1-infected patients resistant to other drug classes. In order to assess its central nervous system penetration, we measured raltegravir concentrations in cerebrospinal fluid (CSF) and plasma in subjects receiving antiretroviral treatment regimens containing this drug.

**Methods:**

Raltegravir concentrations were determined by liquid chromatography tandem mass spectrometry in 25 paired CSF and plasma samples from 16 HIV-1-infected individuals. The lower limit of quantitation was 2.0 ng/ml for CSF and 10 ng/ml for plasma.

**Results:**

Twenty-four of the 25 CSF samples had detectable raltegravir concentrations with a median raltegravir concentration of 18.4 ng/ml (range, <2.0–126.0). The median plasma raltegravir concentration was 448 ng/ml (range, 37–5180). CSF raltegravir concentrations correlated with CSF:plasma albumin ratios and CSF albumin concentrations.

**Conclusions:**

Approximately 50% of the CSF specimens exceeded the IC_95_ levels reported to inhibit HIV-1 strains without resistance to integrase inhibitors. In addition to contributing to control of systemic HIV-1 infection, raltegravir achieves local inhibitory concentrations in CSF in most, but not all, patients. Blood-brain and blood-CSF barriers likely restrict drug entry, while enhanced permeability of these barriers enhances drug entry.

## Introduction

Combination antiretroviral therapy (cART) has markedly reduced the morbidity and mortality of HIV-1 infection, transforming a generally lethal infection into a chronic disease amenable to medical management [1]. Treatment has not only reduced systemic disease, but also the various neurological complications, including both central nervous system (CNS) opportunistic infections and AIDS dementia complex (ADC) associated with HIV-1 encephalitis (HIVE) [2]. These salutary preventative and therapeutic effects on CNS diseases likely result from systemic effects of therapy that maintain host defenses, reduce immune activation and limit continuous re-seeding of the brain [3]. However, more direct therapeutic effects on HIV-1 replication within the CNS may also be important in some patients [4]. This latter effect requires that suppressive levels of drug reach infected cells within the CNS to inhibit local virus propagation.

Raltegravir is the first HIV-1 integrase inhibitor to be licensed for treatment, and is currently indicated for patients with resistance to other classes of drugs [5]. It has been shown to potently reduce plasma viremia without cross-resistance to other licensed drugs [6]. Its pharmacokinetics allows twice daily drug administration. Raltegravir is approximately 83% bound to plasma proteins and eliminated mainly by metabolism via uridine diphosphate glucuronosyltransferase (UGT) 1A1-mediated glucuronidation [7].

We undertook this study in order to assess the pharmacokinetics of raltegravir in the cerebrospinal fluid (CSF) space. For an antiretroviral drug to directly inhibit viral replication in the CNS, it must be able to penetrate the blood-brain barrier (BBB) [8]. The capacity of a drug to enter the CNS depends on a number of factors: molecular size, lipophilicity, degree of ionization and plasma protein binding, and whether or not the drug is a substrate for transmembrane transporters such as multidrug resistance P-glycoprotein (P-gp) [9]. While the CSF space is not synonymous with the brain extracellular environment, it is a convenient surrogate to measure drug penetration and antiviral effects across the BBB and blood-CSF barrier (BCB) [10]. We therefore measured raltegravir concentrations in CSF and plasma in a series of subjects receiving this drug and undergoing lumbar punctures (LPs) in the context of protocols examining other aspects of CNS HIV-1 infection.

## Materials and Methods

Two groups of subjects at our two institutions who were participating in studies involving LPs were included in the study: subjects taking raltegravir as part of their therapeutic regimen for resistant HIV-1 infection, and subjects participating in a treatment intensification study adding raltegravir to their already-suppressive (plasma HIV-1 RNA concentrations <40 copies/ml) regimens. Both groups received raltegravir at the recommended therapeutic dosage (400 mg by mouth twice daily). Treatment adherence was monitored only by diaries, pill counts and questioning of subjects, but not by more direct methods. CSF and blood samples were obtained under the auspices of research protocols approved by the ethics committees of both sites, and all subjects gave their informed consent. LPs were conducted in a standardised manner, and CSF and plasma specimens were clarified by centrifugation and immediately stored at −70°C until analysis.

Total raltegravir concentrations in plasma and CSF were measured by liquid chromatography tandem mass spectrometry (LC-MS/MS) using ^13^C-labeled raltegravir as the internal standard. One hundred µl aliquots of plasma or CSF were treated with acetonitrile to precipitate proteins and thereafter centrifuged at 20,000 g. Ten µl of the supernatants were injected onto the high performance liquid chromatography (HPLC) column (5 cm×2.1 mm Zorbax C-8, 5 mm column). Calibrators in plasma (10 to 2400 ng/ml of raltegravir) and in CSF (2.0 to 100 ng/ml of raltegravir) were analyzed with all samples, along with quality assurance controls at low, medium, and high concentrations. The LC-MS/MS system used was an API 2000 (Applied Biosystems, Foster City, CA) triple-quadruple mass spectrometer coupled to twin Micro-LC pumps and autosampler (Perkin Elmer, Waltham, MA). The lower limit of quantitation (LLQ) was 10 ng/ml for plasma and 2.0 ng/ml for CSF, with coefficients of variation ranging from 3.6% to 6.95% for plasma and 4.9% to 6.3% for CSF. Acceptance criteria for the bioanalytical runs were in line with current Food and Drug Administration (FDA) guidance on bioanalysis with respect to accuracy and precision on calibrators and QA samples. CSF values below the LLQ are presented as 1.0 ng/ml for descriptives.

HIV-1 RNA was quantified in CSF and plasma with COBAS Amplicor HIV-1 Monitor Test, version 1.5 (Roche AB, Basel, Switzerland) (Gothenburg samples) or Abbott RealTime HIV-1 (Abbott Molecular Inc, Des Plaines, IL) (San Francisco samples), and run according to the manufacturers' protocols. These assays have lower detection limits of 20 copies/ml or less, and all values below this are presented as 19 copies/ml (1.28 log_10_ copies/ml) for descriptives. Blood CD4^+^ T-cell count, CSF and blood albumin, and CSF cell counts were performed in the local clinical laboratories using standard methods. CSF:blood albumin ratios (calculated as CSF albumin (ng/ml)/blood albumin (g/l)) were used as an index of BBB/BCB disruption [11].

Nonparametric methods were used for group descriptives and statistical correlations unless otherwise indicated. Pharmaco kinetic estimates used area under the plasma or CSF concentration versus time curve (AUC) to compare exposures in the two fluids. These analyses used Prism 5 (Graphpad, San Diego, CA).

## Results

### Subjects

Ten resistant and six intensification subjects were included in the study, providing a total of 25 visits with CSF and plasma measurements of raltegravir: 15 from San Francisco (including all in the intensification study) and 10 from Gothenburg (Table 1). All were male except two in the resistant group. The ages were similar in the two groups, but blood CD4 counts were lower in the resistant group. Because the HIV-1 RNA measurements in the intensification study will be run in batch after enrollment is completed, these results are not yet available. CSF was generally non-inflammatory, though eight subjects had elevated WBC counts (seven in the treatment group with WBC counts between 6 and 22 cells/µl and one in the intensification group with 8 cells/µl). Group values of both CSF albumin and the calculated CSF:plasma albumin ratios were mildly elevated compared to published normal values (albumin means for 41–50 year olds 20.4 mg/dl, SD 5.7 mg/dl and for 51–60 year olds 24.2 mg/dl mean and 7.6 mg/dl SD; mean albumin ratios 4.6 with SD of 1.3 for 41–50 year olds and 5.5 with SD of 1.7 for 51–60 year olds [11]). The duration of raltegravir treatment ranged from 4 days in one subject to 88 weeks, with an overall median of 11 weeks of treatment. The most common accompanying antiviral drugs included two nucleoside reverse transcriptase inhibitors (NRTIs) and one protease inhibitor (PI).

**Table 1:** Subject Characteristics.

	Resistant	Intensification	Total
	***number***		
Subjects	10	6	16
Sampling Visits	15	10	25
Male∶Female	8∶2	6∶0	14∶2
	***median (range)***	***median (range)***	***median (IQR)***
Age	45 (22–63)	51 (48–62)	49 (44–55)
CD4 (×10^6^/l)	140 (0–490)	388 (198–819)	330 (137–436)
Plasma HIV RNA (log_10_ copies/ml)	2.23 (1.28–5.20)	nd	
CSF HIV RNA (log_10_ copies/ml)	1.28 (1.28–5.66)	nd	
CSF WBC (cells/µl)	4 (1–22)	2 (1–8)	3 (1–6)
CSF Albumin (mg/dl)	22.7 (17.4–54.9)	28.6 (15.3–39.0)	25.5 (18.5–33.3)
CSF:Plasma Albumin Ratio	5.66 (3.78–23.87)	6.21 (3.92–9.29)	5.93 (4.66–7.91)
Time on raltegravir (weeks)	10 (0.5–88)	8 (4–12)	10 (4–25)
***Other Antiretroviral Medications***			
NRTIs	3 (0–3)	3 (2–3)	3 (2–3)
NNRTIs	0 (0–1)	0.5 (0–1)	0 (0–0.5)
PIs	1 (0–1)	1 (0–1)	1 (1–1)
EIs	0 (0–1)	0 (0–0)	0 (0–0)
Total	4 (1–4)	4 (3–5)	4 (3–4)

NRTIs and NNRTIs: nucleoside and non-nucleoside reverse transcriptase inhibitors; PIs: protease inhibitors; EIs: entry inhibitors.

### Raltegravir concentrations

**Figure 1:**
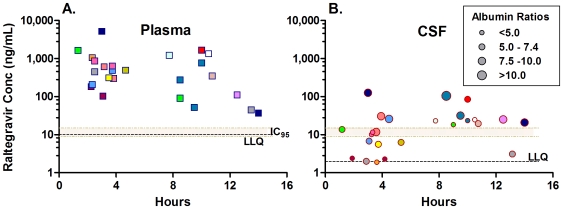
Raltegravir concentrations in plasma (A) and CSF (B) in relation to the time after the previous dose. Results for individual subject evaluations and repeat subject evaluations are indicated by the matching color of squares (plasma) and circles (CSF) coded for each subject. A: The horizontal grey fill plots IC_95_ range of 9.0–15.0 ng/ml, while the lower dashed horizontal line within this zone shows the lower limit of quantitation (LLQ) for plasma of 10.0 ng/ml. B: The one CSF measurement of raltegravir below the LLQ is plotted as if equal to 1.9 ng/ml (orange circle). The size of the symbols in B. also varies with the CSF:albumin ratios at the time of sampling as shown by the grey circles in the key: the largest circles show the one sample with ratio >10, the next largest the 5 samples for albumin ratios from 7.5–10, the next the 10 samples with ratios from 5–7.4, and the smallest the 9 samples for ratios <5. The dashed horizontal line within this zone shows the LLQ for CSF of 2.0 ng/ml.

Figure 1 shows total CSF and plasma raltegravir concentrations in relation to the time of sampling after the previous dose. Samples were obtained at time points following dosing determined by the parent study with two sample times extending beyond the 12 hour dosing interval (median 7.8 hours, range 1.2–14 hours). All of the plasma raltegravir concentrations were above the detection limit (10 ng/ml) and the in vitro IC_95_ (drug concentration needed to inhibit 95% of viral replication) levels from 9.0–15.0 ng/ml [7] and ranged from 37 to 5180 ng/ml (median 448 ng/ml) (Figure 1A). Twenty-four out of 25 CSF samples had detectable raltegravir levels above the limit of detection (2.0 ng/ml) ranging from 2.0 to 126 ng/ml (median 18.4 ng/ml) (Figure 1B). Thirteen of the 25 CSF samples had raltegravir concentrations above 15.0 ng/ml, four were between the reported inhibitory concentrations of 9.0–15.0 ng/ml, and eight were below 9.0 ng/ml. The CSF:plasma ratios for paired samples ranged from 0.01 to 0.61 (median 0.03) in those with detectable CSF raltegravir. The ratio of the estimated CSF:plasma raltegravir AUCs over the dosing interval was 0.03, the same as the median of the CSF:plasma ratios of all the paired samples with measureable CSF raltegravir.

### Relation to CSF albumin concentrations

We explored relationships between the CSF raltegravir concentrations and other measured variables, including the plasma raltegravir concentrations, CSF albumin concentrations, the CSF:plasma albumin ratios, CSF WBC counts and blood CD4+ T cells. Of these, only the related CSF albumin concentrations (P = 0.028, Spearman's r = 0.438) and CSF:blood albumin ratios (P = 0.008, Spearman's r = 0.517) were significant. In Figure 1B the symbol size varies according to the subjects' albumin ratios at the time of sampling, allowing direct visualization of the CSF concentrations in the context of the given albumin ratio ranges.

**Figure 2:**
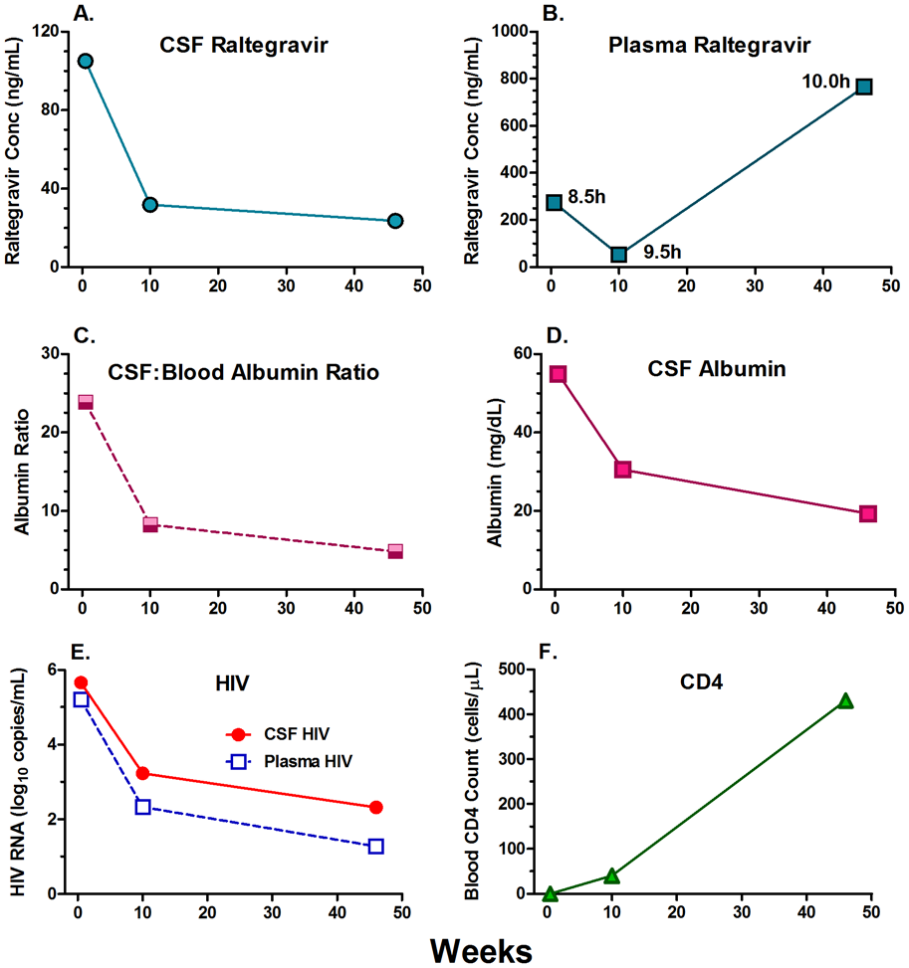
Longitudinal data from patient with three samples over the course of her illness (see text). A: CSF raltegravir concentrations fell as the patient recovered. B: Plasma raltegravir concentrations were variable despite similar times of sampling (shown in hours after dosing adjacent to symbols). C: Decline in the albumin ratio with recovery. D: Similar decline in the CSF albumin concentrations. E: HIV-1 RNA treatment response was parallel in plasma and CSF, with higher levels in the latter. F: Rapid blood CD4+ T cell recovery.

One of the subjects had three LPs during the course of her illness and appears to illustrate the effect of altered BBB permeability on CSF raltegravir levels (Figure 2). This patient was 22 years old when she presented with multiple cerebral *Mycobacterium avium* brain abscess. She had previously been treated with several antiretroviral medications and her virus was multiresistant. Hence, raltegravir was included in her regimen which was started at diagnosis, along with treatment of her mycobacterial infection with rifabutin, azithromycine and ethambutol. She responded very well to these treatments. As shown in Figure 2A, her initial CSF raltegravir was relatively high (105 ng/ml), while the subsequent levels were lower (31.8 and 23.5 ng/ml). This variation in drug concentrations did not correlate with changes in plasma raltegravir levels (Figure 2B), despite all 3 samplings being taken at a similar, relatively late time in the dosing interval (8.5–10 hours). By contrast, the pattern of reduction in the high baseline CSF:plasma albumin ratio (Figure 2C) and CSF albumin concentration (Figure 2D) paralleled the CSF raltegravir concentration, suggesting that the lower CSF drug concentration was related to restoration of the BBB/BCB as the patient recovered clinically with reduction of plasma and CSF HIV-1 RNA levels (Figure 2E) and rapid increase in blood CD4+ T cells (Figure 2F), along with overall clinical improvement.

## Discussion

While detected in all but one subject, total (i.e. bound and unbound) raltegravir CSF concentrations were considerably less than those of plasma, overall estimated at about three percent. While plasma drug concentrations were quite variable as previously described [12], they all were in the therapeutic range. The CSF concentrations also varied, but were both above and below in vitro estimates of inhibitory drug concentrations.

In individual cases it appeared that higher plasma raltegravir concentrations were associated with higher CSF concentrations, but this was not entirely consistent. Indeed, fully evaluating the impact of different factors determining CSF exposure (plasma concentration, time of sample collection following dose, BBB dysfunction, concomitant drug administration, etc.) is complex and will require a larger sample and more detailed modeling than allowed by this relatively small study. Previous studies show that plasma raltegravir levels vary, but display biphasic decay with a terminal half-life of 7–12 hours, reach steady state within two days, and achieve concentrations similar to those in this study [7]. Our observations suggest that CSF raltegravir concentrations are also variable, and while plasma raltegravir and its decay kinetics are likely important to achieving therapeutic CSF levels, other factors also appear to be important.

In theory, it is the unbound fraction of an antiretroviral drug that determines antiviral effect and it is also this fraction that is available to traverse biological membranes and enter target tissue compartments and cells. If one estimates the free concentration of raltegravir in plasma in this study at 76 ng/ml (17% of the median total concentration, 448 ng/ml), and if unbound raltegravir diffused passively into the CSF in the absence of any active transport, the CSF drug concentrations should have been approximately equivalent to this unbound plasma level. However, the median total CSF raltegravir levels were nearly 4–fold lower. Even ignoring the lower binding in CSF, this suggests limited drug entry or its active transport out of the CSF. Indeed, raltegravir has previously been suggested to be a substrate for P-gp transport [13].

Correlation of CSF raltegravir concentrations with the albumin ratios and CSF albumin concentrations, which provide measures of BBB/BCB permeability, also suggests that these barriers are active in restricting raltegravir entry. The illustrated case (Figure 2) with three LPs during the course of her recovery from CNS infection also presents anecdotal supporting evidence. As her albumin ratio decreased from a very high value of 23.9, her CSF raltegravir levels decreased in close parallel despite an increase in plasma raltegravir on the third sampling. This case also underscores the mixed interactions of raltegravir with the BBB and albumin itself. On the one hand, the albumin ratio serves as an indicator of the BBB disruption that likely enhances entry of raltegravir. On the other hand, since albumin also binds raltegravir, the free level of drug will also decrease proportionally for a given total drug level as the albumin increases. Also, since albumin levels in CSF are about 5 percent of those in blood, the bound concentration in CSF is much lower than that in blood and contributes less to the total measured drug. We did not directly measure the unbound drug fraction in either plasma or CSF.

A salient question is whether the CSF raltegravir concentrations are sufficient to directly inhibit local CNS HIV-1 replication. Here again, one must consider the estimated free and bound raltegravir in CSF, and the extracellular concentrations to achieve inhibition of viral replication. Furthermore, treatment of HIVE that is sustained in perivascular and parenchymal macrophages and microglia relies on the drug concentration in brain extracellular fluid surrounding these infected cells which may differ from CSF concentration. Current estimates of inhibitory concentrations depend largely on cell culture experiments using varying amounts of human or fetal serum that bind raltegravir. Published values for the IC_95_ are 15.0 ng/ml (33 nM) obtained in the presence of 50% human serum and approximately 9.0 ng/ml (18.7 nM) measured with 10% fetal bovine serum [7], [14]; these are the figures used here to estimate in vivo effectiveness. Although CSF is likely to bind less of the drug, if one takes these values as spanning the lower range of effectiveness, eight of the CSF samples in this study had lower raltegravir levels (<9.0 ng/ml), four had levels between 9.0–15.0 ng/ml, and thirteen had higher concentrations (Figure 1). This suggests that raltegravir CSF exposure was likely inhibitory in the CSF in more than half of the cases, exceeding or within the spectrum of the IC_95_ of non-resistant virus. However, in some patients CSF drug levels may well have been too low for this direct suppressive activity.

In conclusion, raltegravir may directly contribute to treatment of the autonomous component of CNS HIV-1 infection that is sustained locally in some, but not all patients. Hence, its use in the setting of ADC/HIVE or when direct CNS antiviral effect is considered critical should be accompanied by careful individual patient monitoring, including assessment of CSF HIV RNA responses. Increases in BBB/BCB permeability associated with HIVE [15] may actually favorably influence the treatment effect of raltegravir in the setting of overt ADC as it seemed to in the described patient. While we consider these results encouraging with respect to inclusion of raltegravir in treatment regimens targeting CNS infection, they also suggest the need for accompanying CNS-penetrating drugs in this setting, and indicate the need for more direct study of raltegravir CNS or CSF antiviral effects to address this question. It is also important to emphasize that antiviral drug impact on CNS infection involves other factors that relate to their systemic effects, including preservation or restoration of host defenses against HIV-1, control of immune activation that contributes to CNS infection, and prevention of continued seeding of the CNS [16].
